# A prognostic nomogram for stage II/III rectal cancer patients treated with neoadjuvant chemoradiotherapy followed by surgical resection

**DOI:** 10.1186/s12893-022-01710-z

**Published:** 2022-07-04

**Authors:** Yanfei Lin

**Affiliations:** Department of General Surgery, Xiamen Branch, Zhongshan Hospital, Fudan University, Jinhu Road 668, Huli District, Xiamen, 361015 China

**Keywords:** Stage II/III rectal cancer, Neoadjuvant chemoradiotherapy, Surgical resection, Nomogram, Log odds of positive nodes (LODDS)

## Abstract

**Background:**

The purpose of this study was to develop a large population-based nomogram incorporating the log odds of positive nodes (LODDS) for predicting the overall survival (OS) of stage II/III rectal cancer patients treated with neoadjuvant chemoradiotherapy (NCRT) followed by surgical resection.

**Methods:**

The Surveillance, Epidemiology, and End Results database was used to collect information on patients diagnosed with stage II/III rectal cancer between 2010 and 2015 and treated with NCRT followed by surgical resection. The Cox regression analyses were performed to determine the independent prognostic factors. In this study, LODDS was employed instead of American Joint Committee on Cancer (AJCC) 7th N stage to determine lymph node status. Then a nomogram integrating independent prognostic factors was developed to predict the 24-, 36-, and 60-month overall survival. The receiver operating characteristic (ROC) curves and calibration curves were used to validate the nomogram. Furthermore, patients were stratified into three risk groups (high-, middle-, and low-risk) based on the total points obtained from the nomogram. And Kaplan–Meier curves were plotted to compare the OS of the three groups.

**Results:**

A total of 3829 patients were included in the study. Race, sex, age, marital status, T stage, tumor grade, tumor size, LODDS, CEA level, and postoperative chemotherapy were identified as independent prognostic factors, based on which the prognostic nomogram was developed. The area under curve values of the nomogram for the 24-, 36-, and 60-month OS in the training cohort were 0.736, 0.720, and 0.688, respectively; and 0.691, 0.696, and 0.694 in the validation cohort, respectively. In both the validation and training cohorts, the calibration curves showed a high degree of consistency between actual and nomogram-predicted survival rates. The Kaplan–Meier curves showed that the three risk groups had significant differences in overall survival (P < 0.001).

**Conclusion:**

A large population-based nomogram incorporating LODDS was developed to assist in evaluating the prognosis of stage II/III rectal cancer patients treated with NCRT followed by surgical resection. The nomogram showed a satisfactorily discriminative and stable ability to predict the OS for those patients.

**Supplementary Information:**

The online version contains supplementary material available at 10.1186/s12893-022-01710-z.

## Introduction

For patients with locally advanced rectal cancer (LARC), neoadjuvant chemoradiotherapy (NCRT) followed by surgical resection has become the standard therapy [1]. NCRT can help to downstage tumors, increase the rate of curative resection, and decrease the rate of local recurrence [2]. According to data from the Surveillance, Epidemiology, and End Results (SEER) database, about 35.7% of patients with rectal cancer in stage II/III received NCRT [3]. The reason could be that the survival benefit of NCRT has so far been controversial [4, 5], and radiotherapy can make surgical dissection more difficult and increase the risk of postoperative complications, such as anastomotic leakage, wound infection, and pelvic abscess [6]. Patients with stage II/III rectal cancer who were treated with NCRT followed by surgical resection form a distinct cohort with specific prognosis characteristics.

Nomograms have been routinely utilized to predict cancer outcomes In recent years [7]. A nomogram integrating multiple prognostic factors can be used to estimate the probability of a particular result. Useful in individualized medicine, it has been applied to a wide range of malignancies [8, 9], For patients with rectal cancer, a number of nomograms have been developed to date. However, nomograms for stage II/III rectal cancer patients treated with NCRT followed by surgical resection have rarely been reported. The aim of this study was to utilize the SEER database to develop a nomogram to predict these patients' prognosis.

The log odds of positive nodes (LODDS) was defined as the log of the ratio between the number of positive lymph nodes (PLN) and negative lymph nodes (NLN) [10]. In recent years, it has been proposed and confirmed as a reliable indicator of prognosis in a variety of cancers, such as pancreatic cancer [11], colorectal cancer [12], and gastric cancer [13], and has proven to be a superior predictive factor in patients with colorectal cancer when compared to the American Joint Committee on Cancer (AJCC) N stage [14, 15]. As a result, instead of AJCC N stage, I incorporated LODDS into this nomogram and expected this nomogram can assist in evaluating the prognosis of stage II/III rectal cancer patients treated with NCRT followed by surgical resection.

## Methods

### Patient selection

**Fig. 1:**
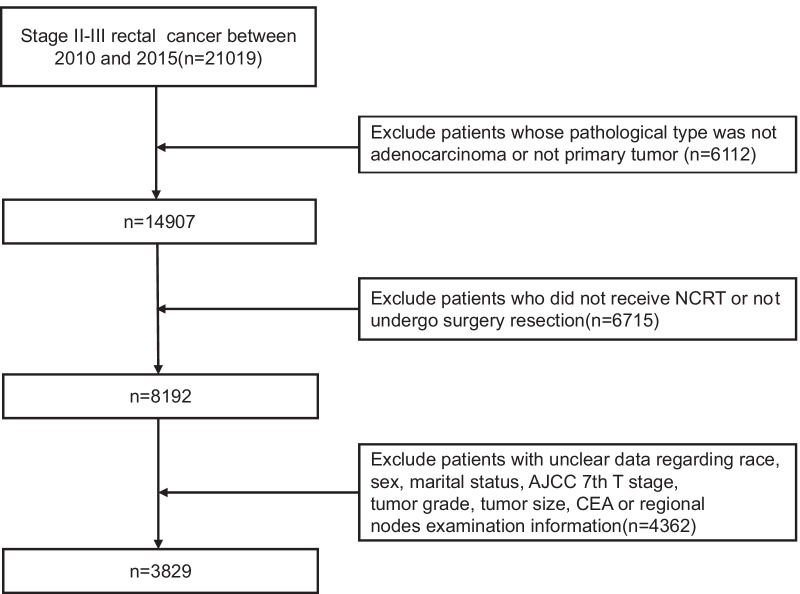
Flow diagram of the study selection process. *NCRT* neoadjuvant chemoradiotherapy, *CEA* carcinoembryonic antigen, *AJCC* American Joint Committee on Cancer

This population-based study was conducted using the SEER database. The SEER database collects statistical, oncological, diagnostic, treatment, and survival information from specific geographical regions representing roughly 28% of the US population. Database was obtained from the SEER*Stat software (version 8.3.6). Patients who were diagnosed after 2015 were not included in this study to ensure enough time for follow-up. In this study, only patients with stage II/III rectal cancer diagnosed between 2010 and 2015 were included. The following were the criteria for inclusion: (1) rectal cancer as the only primary tumor; (2) histologically confirmed as rectal adenocarcinoma; (3) stage II/III rectal cancer patients treated with NCRT followed by surgical resection; and (4) patients with complete survival information, demographic data, and clinicopathologic features. The data selection procedure is shown in Fig. 1. Finally, a total of 3829 patients were identified. At a 7:3 ratio, study patients were randomly assigned to the training cohort and validation cohort.

### Statistical analysis

SPSS 26.0 was used for statistical analysis. The proportions between the training cohort and validation cohort were compared using Pearson’s Chi-square test. The best tumor size cutoff was calculated using X-tile software [16], and tumor size was grouped as < 4.6, 4.6–7.0, and ≥ 7.0 cm. LODDS was calculated using the following formula: LODDS = log [(0.5 + PLN)/(0.5 + NLN)] [17]. A P value of less than 0.05 was considered statistically significant. The univariate and multivariate Cox regression analyses were performed to determine the independent prognostic factors. The hazard ratio (HR, 95% confidence interval (CI)) was used to express the risk factors. A nomogram integrating independent prognostic factors was developed to predict the 24-, 36-, and 60-month overall survival of patients and conducted with R software (version 4.1.0). Receiver operating characteristic (ROC) curves were constructed and the area under curve values (AUCs) were determined to validate the discrimination of nomograms. To assess the consistency between actual and nomogram-predicted survival rates, calibration curves were plotted. Furthermore, X-tile software were used to stratify patients into three risk groups (high-, middle-, and low-risk) according to the total points obtained from the nomogram. And Kaplan–Meier curves were plotted to compare OS of the three groups.

## Results

### Clinicopathologic and demographic characteristics

3829 rectal cancer patients were included and randomly assigned in a 7:3 ratio to a training cohort (n = 2678) and validation cohort (n = 1151) (see Additional file 1 and Additional file 2). Table 1 showed the patients’ clinicopathologic and demographic characteristics and no statistically significant differences were found between the two cohorts. Overall, patients of white ethnicity (80.9%) made up the majority of the entire population, and men (62.9%) accounted for more than half of the patients. Tumor grade II was the most common (80.6%). The majority of patients were classified as T3 by the AJCC (7th edition) tumor-node-metastasis (TNM) system (83.3%). LODDS between − 1.5 and − 0.4 were found in roughly 62.5 percent of patients. More than half (56.9%) of the patients received postoperative chemotherapy and only a small percentage of patients (3.4%) underwent postoperative radiotherapy.

**Table 1:** Demographic and clinicopathologic characteristics of the included patients

Variables	Total number (n = 3829), n (%)	Training cohort (n = 2678), n (%)	Validation cohort (n = 1151), n (%)	P
Age				
< 60	2120 (55.4)	1493 (55.7)	627 (54.5)	0.466
≥ 60	1709 (44.6)	1185 (44.3)	524 (45.5)	
Race				
White	3096 (80.9)	2176 (81.2)	920 (79.9)	0.624
Black	289 (7.5)	199 (7.4)	90 (7.8)	
Other^†^	444 (11.6)	303 (11.3)	141 (12.4)	
Sex				
Female	1419 (37.1)	988 (36.9)	431 (37.4)	0.746
Male	2410 (62.9)	1690 (63.1)	720 (62.6)	
Marital status				
No^‡^	1540 (40.2)	1082 (40.4)	458 (39.8)	0.723
Yes	2289 (59.8)	1596 (59.6)	693 (60.2)	
Grade				
I	282 (7.4)	194 (7.2)	88 (76.5)	0.895
II	3088 (80.6)	2157 (80.5)	931 (80.9)	
III	408 (10.6)	290 (10.8)	118 (10.3)	
IV	51 (1.3)	37 (1.4)	14 (1.2)	
T stage				
T1	33 (0.9)	26 (1.0)	7 (0.6)	0.789
T2	181 (4.7)	124 (4.6)	57 (5.0)	
T3	3190 (83.3)	2233 (83.4)	957 (83.1)	
T4a	108 (2.8)	77 (2.9)	31 (2.7)	
T4b	317 (8.3)	218 (8.1)	99 (8.6)	
LODDS				
< − 1.5	1020 (26.6)	714 (26.7)	306 (26.6)	0.521
− 1.5 ~ − 0.4	2394 (62.5)	1664 (62.1)	730 (63.4)	
≥ − 0.4	415 (10.8)	300 (11.2)	115 (10.0)	
Tumor size(mm)				
< 46	1942 (50.7)	1360 (50.8)	582 (50.6)	0.747
46–70	1349 (35.2)	949 (35.4)	400 (34.7)	
≥ 70	538 (14.1)	369 (13.8)	169 (14.7)	
CEA level				
Negative	2144 (56.0)	1514 (56.5)	630 (54.7)	0.304
Positive	1685 (44.0)	1164 (43.5)	521 (45.3)	
Postoperative chemotherapy				
No	1651 (43.1)	1166 (43.5)	485 (42.1)	0.422
Yes	2178 (56.9)	1512 (56.5)	666 (57.9)	
Postoperative radiation				
No	3700 (96.6)	2593 (96.8)	1107 (96.2)	0.308
Yes	129 (3.4)	85 (3.2)	44 (3.8)	

^†^Including American Indian/AK Native, Asian/Pacific Islander; ^‡^Including Widowed, Divorced, Single and Unmarried or Domestic Partner, Separated; *CEA* carcinoembryonic antigen, *LODDS* log odds of positive lymph nodes

### Prognostic factors associated with OS

To determine the prognostic factors, the univariate and multivariate analyses were conducted included the patients from the training cohort. As shown in Table 2 in the univariate analysis, Race, sex, age, marital status, T stage, tumor grade, tumor size, LODDS, CEA level, and postoperative chemotherapy were statistically associated with OS (*P* < 0.05). The effects of postoperative radiation were not statistically significant (*P* > 0.05). Finally, race, sex, age, marital status, T stage, tumor grade, tumor size, LODDS, CEA level, and postoperative chemotherapy were all revealed to be independent predictive variables in the multivariate analysis. Advanced age, male sex, unmarried status, higher tumor grade, elevated CEA levels, and tumor size greater than 70 mm was related to a higher risk of mortality. T4b and LODDS greater than − 0.4 were obviously detrimental to patient survival. Furthermore, as measured by a hazard ratio of 0.81 (95% CI: 0.693–0.947), patients who underwent postoperative chemotherapy showed a reduced mortality rate.

**Table 2:** Univariate and multivariate Cox analyses in II/III rectal cancer patients treated with NCRT followed by surgical resection

Variable	Univariate analysis			Multivariate analysis		
	HR	95% CI	P	HR	95% CI	P
Age						
< 60	Reference			Reference		
≥ 60	1.282	1.094–1.502	0.002**	1.344	1.145–1.579	< 0.001***
Race						
Black	Reference			Reference		
White	0.682	0.531–0.877	0.003**	0.666	0.517–0.858	0.002**
Other	0.719	0.522–0.900	0.044*	0.682	0.492–0.944	0.021*
Sex						
Female	Reference			Reference		
Male	1.282	1.094–1.502	0.002**	1.344	1.145–1.579	< 0.001***
Marital status						
No	Reference			Reference		
Yes	0.790	0.680–0.916	0.002**	0.829	0.711–0.966	0.016*
Grade						
I	Reference			Reference		
II	1.081	0.797–1.465	0.618	1.129	0.831–1.534	0.438
III	1.718	1.212–2.436	0.002**	1.567	1.098–2.235	0.013*
IV	1.891	1.038–3.446	0.037	1.815	0.985–3.343	0.056
T stage						
T1	Reference			Reference		
T2	1.312	0.455–3.783	0.615	1.481	0.507–4.331	0.473
T3	1.730	0.647–4.625	0.275	1.866	0.688–5.064	0.221
T4a	2.360	0.821–6.783	0.111	2.252	0.774–6.550	0.136
T4b	3.254	1.194–8.870	0.021*	3.141	1.136–8.681	0.027*
LODDS						
< − 1.5	Reference			Reference		
− 1.5 ~ − 0.4	1.660	1.355–2.034	< 0.001***	1.678	1.368–2.059	< 0.001***
≥ − 0.4	3.466	2.716–4.423	< 0.001***	3.616	2.820–4.637	< 0.001***
Tumor size (mm)						
< 46	Reference			Reference		
46–70	1.050	0.889–1.239	0.565	0.994	0.840–1.177	0.946
≥ 70	1.528	1.243–1.878	< 0.001***	1.309	1.056–1.623	0.014*
CEA level						
Negative	Reference			Reference		
Positive	1.567	1.351–1.817	< 0.001***	1.384	1.189–1.612	< 0.001***
Postoperative chemotherapy						
No	Reference			Reference		
Yes	0.797	0.685–0.928	0.003**	0.810	0.693–0.947	0.008**
Postoperative radiation						
No	Reference					
Yes	1.397	0.969–2.013	0.073			

*P < 0.05; **P < 0.01; ***P < 0.001. *NCRT* neoadjuvant chemoradiotherapy, *HR* hazard ratio, *CEA* carcinoembryonic antigen, *LODDS* log odds of positive lymph nodes

### Development and validation of prognostic nomogram

**Fig. 2:**
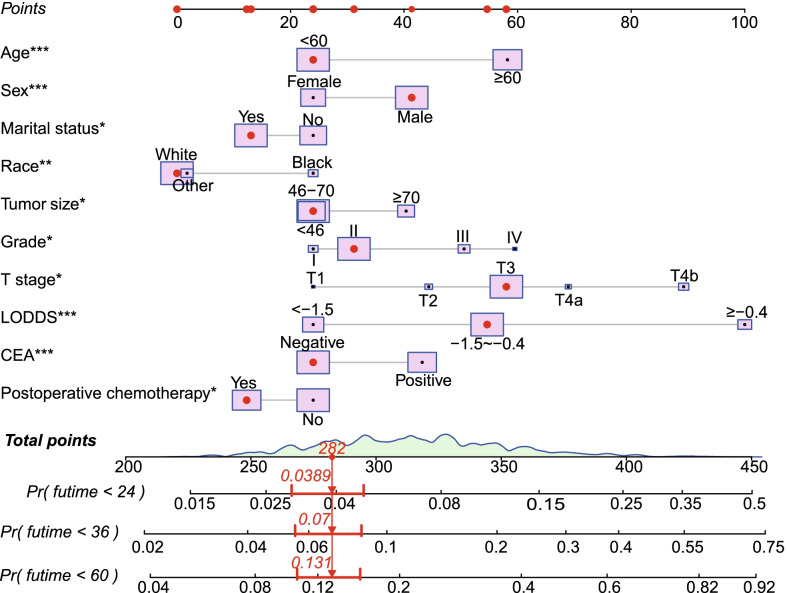
Nomogram for predicting the OS at 24, 36 and 60 months. According to Nomogram’s estimate the cumulative risk of OS in patients no.30 at 24, 36 and 60 months is 0.0389, 0.07 and 0.131, respectively. *P < 0.05; **P < 0.01; ***P < 0.001. *OS* overall survival, *CEA* carcinoembryonic antigen, *LODDS* log odds of positive lymph nodes

A prognostic nomogram integrating independent prognositc factors for OS was developed (Fig. 2). The predicted probability of 24-, 36-, and 60-month OS could be calculated by summing the points on the nomogram for each patient's matching factors. Figure 2 displayed the survival probability of a given patients was calculated using the nomogram.

**Fig. 3:**
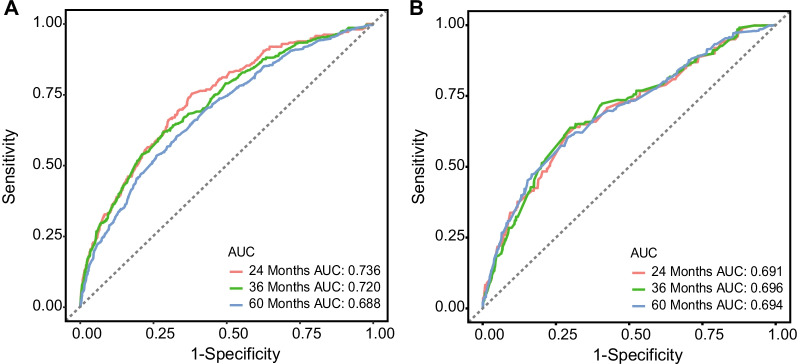
ROC curves and AUC values of the nomogram for the predicted 24-, 36-, and 60-month OS rates for the training cohort (**A**–**C**) and the validation cohort (**D**–**F**). *AUC* area under the curve, *ROC* receiver operating characteristic, *OS* overall survival

**Fig. 4:**
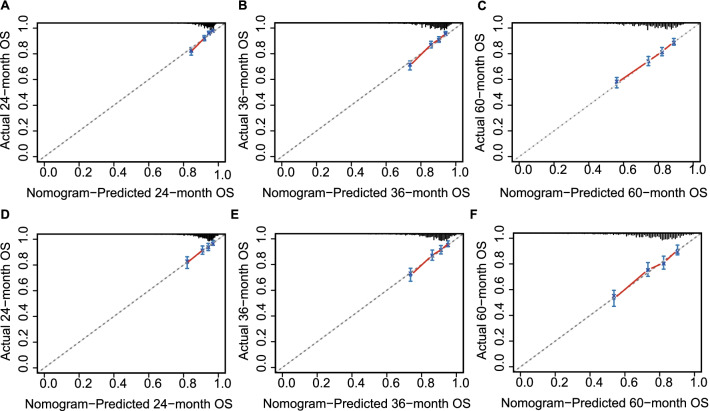
The calibration curves predicting 24-, 36-, 60-month OS in the training cohort (**A**–**C**) and the validation cohort (**D**–**F**). The dashed line represents a perfect match between the nomogram predicted probability (x-axis) and the actual probability calculated. *OS* overall survival

In the validation of the nomogram, the AUCs of the nomogram for the 24-, 36-, and 60-month OS in the training cohort were 0.736, 0.720, and 0.688, respectively; and 0.691, 0.696, and 0.694 in the validation cohort, respectively. The AUCs demonstrated the satisfactory discriminative power of the model (Fig. 3). In both the validation and training cohorts, the calibration curves showed a high degree of consistency between actual and nomogram-predicted survival rates (Fig. 4).

### Stratification of risk groups

**Fig. 5:**
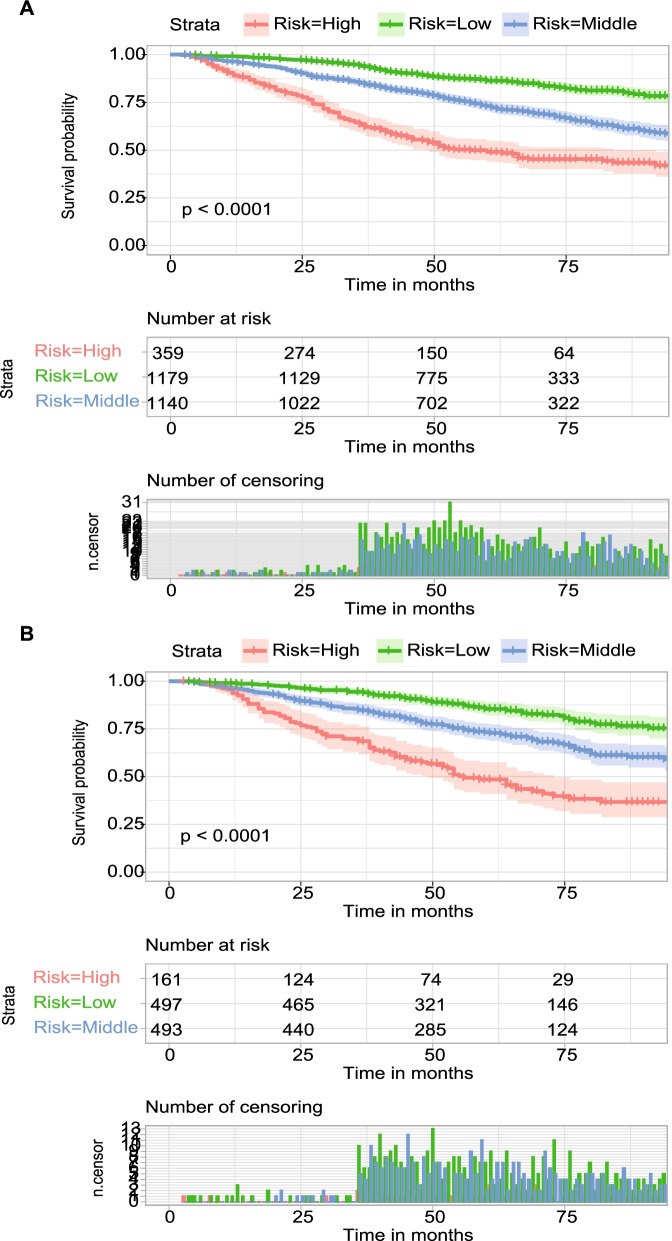
Kaplan–Meier overall survival curves of patients at three risk groups in the training cohort (**A**) and validation cohort (**B**) according to the nomogram

All patients were stratified into three risk groups based on the total points obtained from the nomogram using X-tile software: high-risk (total points ≥ 368), middle-risk (339 ≤ total points < 368), and low-risk (total points < 339). As shown in Fig. 5, the Kaplan–Meier curves indicated that the three risk groups had significant differences in overall survival (P < 0.001). The 5-year OS in the training cohort for the high-, middle-, and low-risk were 49.0% (95% CI: 46.2–51.8%), 73.0% (95% CI: 71.6–74.4%), 86.4% (95% CI: 85.3–87.5%), respectively; and 48.5% (95% CI: 44.3–52.7%), 73.2% (95% CI: 71.0–75.4%), and 85.4% (95% CI: 83.6–87.2%) in the validation cohort, respectively. The mortality rates of patients in the high-risk group were significantly higher than those in the low-risk group.

## Discussion

In this study, the data from 3829 patients in the SEER database were used to develop a prognostic nomogram. Currently, few prognostic nomograms for stage II/III rectal cancer patients treated with NCRT followed by surgical resection have been developed, especially based on a large population. The study by Zhifang et al. covering 785 cases [18], had certain limitations as it was a single-center study and their results lacked externally validation. The analysis conducted by Valentini et al. using data from five European randomized clinical trials was the only nomogram for those patients based on a large population [19], but it did not include other variables that may affect prognosis, such as race, marital status, tumor size, CEA level, and tumor grade. In addition to age, sex, T stage and postoperative chemotherapy, this investigation also identified race, marital status, tumor size, CEA level, tumor grade, and LODDS as independent prognostic factors through Cox regression analysis. All these risk factors were incorporated into the nomogram to improve its preciseness. Besides, to the best of my knowledge, this is the first nomogram that incorporates LODDS to predict the OS of stage II/III rectal cancer patients treated with NCRT followed by surgical resection. Through multivariable Cox regression analysis, black patients may have worse overall survival rates. The findings were consistent with earlier research showing that compared to white patients, black patients are diagnosed at a later stage, receive less benefit from adjuvant chemotherapy, and are more likely to suffer 5-FU toxicity than Whites, probably due to differences in dihydropyridine dehydrogenase status [20, 21]. This study found that patients who were married had better outcomes. According to previous studies, spouses may encourage patients to check their health state, and married cancer patients were diagnosed at an earlier stage than unmarried cancer patients [22]. Furthermore, married patients had better adherence to treatment than unmarried patients, such as receiving NCRT [23]. My results confirmed, for the first time, that tumor size greater than 70 mm was an independent prognostic factor for stage II/III rectal cancer patients treated with NCRT followed by surgical resection. However, recent research shows that tumor volume is superior to tumor size as a prognostic classifier in rectal cancer [24]. In addition, Yeo et al. discovered that the rate of tumor volume reduction in LARC patients receiving NCRT was a significant prognostic factor [25]. However, due to the lack of tumor volume data in the SEER database, this study cannot further confirm these observations.

As we all know, lymph node status plays a significant role in rectal cancer patients’ prognosis. In recent years, in addition to AJCC N stage, many scholars have proposed and analyzed other parameters to validate the status of lymph nodes, such as LODDS and the lymph node ratio (LNR). Although AJCC N stage is currently the most widely used method for lymph node classification, many researchers have pointed out its limitation since it only considers the number of positive lymph nodes and ignores the total number of lymph nodes (TLN) retrieved during surgery. In rectal cancer, TLN has been proven to be an independent prognosis-related factor [26–28]. For accurate staging of colorectal cancer, removal of at least twelve lymph nodes was recommended by AJCC. LNR involves both TLN and PLN and has been claimed to be more accurate than AJCC N stage for rectal cancer [29, 30]. However, other scholars have noticed that when TLN are either all non-metastatic or all metastatic, patients with the same LNR values might be extremely varied. LODDS, as a novel lymph node classification, can overcome the drawbacks of LNR and increase the accuracy for prognostic validation, and studies have shown its superiority to LNR in rectal cancer [17, 31, 32]. For LARC, LODDS was confirmed to perform better than both AJCC N stage and LNR in terms of prognostic predictive value [14, 15, 33]. Therefore, the nomogram will be more convincing and effective if LODDS is incorporated into it. The nomogram clearly demonstrated that LODDS was the most influential factor in a patient’s poor prognosis.

In this study, 56.9% of patients who received postoperative chemotherapy, seemed to have a better outcome. On the other hand, several large randomized controlled trials concluded that postoperative chemotherapy could not improve the OS of rectal patients receiving surgery after NCRT [34–36]. These trials, however, had significant limitations, such as poor adherence in the European Organization for Research and Treatment of Cancer trial 22921. Therefore, it remains uncertain if these patients will benefit from postoperative chemotherapy. According to current National Comprehensive Cancer Network (NCCN) recommendations, rectal patients with tumors staged as T3 or N1 and higher should consider postoperative chemotherapy after neoadjuvant chemotherapy [37]. However, experts convened by the European conference on rectal cancer concluded that there is currently insufficient evidence to demonstrate the benefits of receiving adjuvant chemotherapy after preoperative chemoradiotherapy; hence, postoperative chemotherapy cannot be recommended [38]. In this way, more comprehensive and conclusive researches will be conducted in the future.

In this study, a nomogram was developed to predict the prognosis of II/III rectal cancer patients with treated with NCRT followed by surgical resection. The probability of 24-, 36-, and 60-month OS can be simply calculated by summing the points on the nomogram for each patient's matching factors. The AUCs of the nomogram for the 24-, 36-, and 60-month OS In the training cohort were 0.736, 0.720, and 0.688, respectively. It revealed the discriminative capability of nomogram to predict OS of patients. Through calibration curves, the nomogram showed excellent consistency for predicting 24-, 36-, and 60-year OS. In additional, by employing risk group stratification, patients who are at high risk can be easily tracked down and should be taught to pay closer attention to their health status and have regular follow-up consultations with their doctors. To summarize, this nomogram provides a reliable and discriminative prognostic evaluation for patients with stage II/III rectal cancer treated with NCRT followed by surgical resection.

There are some limitations in this study, first, studies have demonstrated that tumor response to NCRT is one of the most important prognostic factors for LARC patients [39, 40] and that Mandard good responders have a significantly higher OS rate than Mandard bad responders [41]. In addition, Ann et al. concluded that all tumor-response gradings were related to survival and downstaging had an independent effect on OS [42]. However, the lack of a SEER database on tumor response to NCRT, response assessment, and waiting time between radiotherapy and surgery was the study's major limitation. Second, as this was a retrospective analysis based on the SEER database, there was bound to be some degree of selection bias. Third, the SEER database did not offer certain information that could have affected patient outcomes, such as surgical margins, lymph/vascular or perineural invasion, chemotherapy/radiation regimen, exact type of surgical resection, indications to receive postoperative chemotherapy, and complications of NCRT. A more advanced prognostic model is required to assist in evaluating the prognosis of stage II/III rectal cancer patients treated with NCRT followed by surgical resection.

## Conclusion

A large population-based nomogram incorporating LODDS was developed to assist in evaluating the prognosis of stage II/III rectal cancer patients treated with NCRT followed by surgical resection. The nomogram showed a satisfactorily discriminative and stable ability to predict OS for those patients. A more advanced prognostic model is required to assist in evaluating the prognosis of those patients.

## Supplementary Information

**Additional file 1:** The data of the training cohort.

**Additional file 2: **The data of the validation cohort.

## Data Availability

The datasets generated during and/or analysed during the current study are available in the SEER database repository, SEER Data & Software (cancer.gov). All data generated or analysed during this study are included in this published article [Additional files].

## Abbreviations

SEER: Surveillance, Epidemiology, and End Results
OS: Overall survival
NCRT: Neoadjuvant chemoradiotherapy
LODDS: Log odds of positive lymph nodes
PLN: The number of positive lymph nodes
NLN: Negative lymph nodes
HR: Hazard ratio
CEA: Carcinoembryonic antigen
AJCC: American Joint Committee on Cancer
AUC: Area under the curve
ROC: Receiver operating characteristic

## References

[1] Benson AB, Venook AP, Al-Hawary MM, Cederquist L, Chen YJ, Ciombor KK (2018). Rectal Cancer, Version 2.2018, NCCN Clinical Practice Guidelines in Oncology. J Natl Compr Canc Netw.

[2] Deidda S, Elmore U, Rosati R, De Nardi P, Vignali A, Puccetti F (2021). Association of delayed surgery with oncologic long-term outcomes in patients with locally advanced rectal cancer not responding to preoperative chemoradiation. JAMA Surg.

[3] Wang R, Zhao D, Liu YJ, Ye C, Qian JR, Dai JN (2019). Prognostic significance of preoperative radiotherapy in stage II and III rectal cancer patients: a Strobe-compliant study of SEER 18 registries database (1988–2011). Neoplasma.

[4] van Gijn W, Marijnen CA, Nagtegaal ID, Kranenbarg EM, Putter H, Wiggers T (2011). Preoperative radiotherapy combined with total mesorectal excision for resectable rectal cancer: 12-year follow-up of the multicentre, randomised controlled TME trial. Lancet Oncol.

[5] Allegra CJ, Yothers G, O'Connell MJ, Beart RW, Wozniak TF, Pitot HC, et al. Neoadjuvant 5-FU or capecitabine plus radiation with or without oxaliplatin in rectal cancer patients: a phase III randomized clinical trial. J Natl Cancer Inst. 2015;107(11). 10.1093/jnci/djv248 PMC4849360 26374429

[6] Yang J, Luo Y, Tian T, Dong P, Fu Z (2022). Effects of neoadjuvant radiotherapy on postoperative complications in rectal cancer: a meta-analysis. J Oncol.

[7] Balachandran VP, Gonen M, Smith JJ, DeMatteo RP (2015). Nomograms in oncology: more than meets the eye. Lancet Oncol.

[8] Yang J, Pan Z, Zhou Q, Liu Q, Zhao F, Feng X (2019). Nomogram for predicting the survival of patients with malignant melanoma: a population analysis. Oncol Lett.

[9] Song K, Song J, Chen F, Lin K, Ma X, Jiang J (2018). Prognostic nomograms for predicting overall and cancer-specific survival of high-grade osteosarcoma patients. J Bone Oncol.

[10] Persiani R, Cananzi FC, Biondi A, Paliani G, Tufo A, Ferrara F (2012). Log odds of positive lymph nodes in colon cancer: a meaningful ratio-based lymph node classification system. World J Surg.

[11] Ramacciato G, Nigri G, Petrucciani N, Pinna AD, Ravaioli M, Jovine E (2017). Prognostic role of nodal ratio, LODDS, pN in patients with pancreatic cancer with venous involvement. BMC Surg.

[12] Ozawa T, Ishihara S, Sunami E, Kitayama J, Watanabe T (2015). Log odds of positive lymph nodes as a prognostic indicator in stage IV colorectal cancer patients undergoing curative resection. J Surg Oncol.

[13] Aurello P, Petrucciani N, Nigri GR, La Torre M, Magistri P, Tierno S (2014). Log odds of positive lymph nodes (LODDS): what are their role in the prognostic assessment of gastric adenocarcinoma?. J Gastrointest Surg.

[14] Scarinci A, Di Cesare T, Cavaniglia D, Neri T, Colletti M, Cosenza G (2018). The impact of log odds of positive lymph nodes (LODDS) in colon and rectal cancer patient stratification: a single-center analysis of 323 patients. Updates Surg.

[15] Shen F, Cui J, Cai K, Pan H, Bu H, Yu F (2018). Prognostic accuracy of different lymph node staging systems in rectal adenocarcinoma with or without preoperative radiation therapy. Jpn J Clin Oncol.

[16] Camp RL, Dolled-Filhart M, Rimm DL (2004). X-tile: a new bio-informatics tool for biomarker assessment and outcome-based cut-point optimization. Clin Cancer Res.

[17] Huang B, Ni M, Chen C, Cai G, Cai S (2017). LODDS is superior to lymph node ratio for the prognosis of node-positive rectal cancer patients treated with preoperative radiotherapy. Tumori.

[18] Zheng Z, Wang X, Liu Z, Lu X, Huang Y, Chi P (2021). Individualized conditional survival nomograms for patients with locally advanced rectal cancer treated with neoadjuvant chemoradiotherapy and radical surgery. Eur J Surg Oncol.

[19] Valentini V, van Stiphout RG, Lammering G, Gambacorta MA, Barba MC, Bebenek M (2011). Nomograms for predicting local recurrence, distant metastases, and overall survival for patients with locally advanced rectal cancer on the basis of European randomized clinical trials. J Clin Oncol.

[20] Jessup JM, Stewart A, Greene FL, Minsky BD (2005). Adjuvant chemotherapy for stage III colon cancer: implications of race/ethnicity, age, and differentiation. JAMA.

[21] Dimou A, Syrigos KN, Saif MW (2009). Disparities in colorectal cancer in African-Americans vs Whites: before and after diagnosis. World J Gastroenterol.

[22] Wang L, Wilson SE, Stewart DB, Hollenbeak CS (2011). Marital status and colon cancer outcomes in US Surveillance, Epidemiology and End Results registries: does marriage affect cancer survival by gender and stage?. Cancer Epidemiol.

[23] DiMatteo MR (2004). Social support and patient adherence to medical treatment: a meta-analysis. Health Psychol.

[24] Jiang Y, You K, Qiu X, Bi Z, Mo H, Li L (2018). Tumor volume predicts local recurrence in early rectal cancer treated with radical resection: a retrospective observational study of 270 patients. Int J Surg.

[25] Yeo SG, Kim DY, Park JW, Oh JH, Kim SY, Chang HJ (2012). Tumor volume reduction rate after preoperative chemoradiotherapy as a prognostic factor in locally advanced rectal cancer. Int J Radiat Oncol Biol Phys.

[26] Washington MK (2008). Colorectal carcinoma: selected issues in pathologic examination and staging and determination of prognostic factors. Arch Pathol Lab Med.

[27] Tan L, Liu ZL, Ma Z, He Z, Tang LH, Liu YL (2020). Prognostic impact of at least 12 lymph nodes after neoadjuvant therapy in rectal cancer: a meta-analysis. World J Gastrointest Oncol.

[28] Xu Z, Berho ME, Becerra AZ, Aquina CT, Hensley BJ, Arsalanizadeh R (2017). Lymph node yield is an independent predictor of survival in rectal cancer regardless of receipt of neoadjuvant therapy. J Clin Pathol.

[29] Zhou D, Ye M, Bai Y, Rong L, Hou Y (2015). Prognostic value of lymph node ratio in survival of patients with locally advanced rectal cancer. Can J Surg Journal canadien de chirurgie.

[30] Kim JY, Chung SM, Choi BO, Lee IK, An CH, Won JM (2012). Prognostic significance of the lymph node ratio regarding recurrence and survival in rectal cancer patients treated with postoperative chemoradiotherapy. Gut Liver.

[31] Huang B, Chen C, Ni M, Mo S, Cai G, Cai S (2016). Log odds of positive lymph nodes is a superior prognostic indicator in stage III rectal cancer patients: a retrospective analysis of 17,632 patients in the SEER database. Int J Surg.

[32] Lee CW, Wilkinson KH, Sheka AC, Leverson GE, Kennedy GD (2016). The log odds of positive lymph nodes stratifies and predicts survival of high-risk individuals among stage III rectal cancer patients. Oncologist.

[33] Petrucciani N, Carra MC, Martinez-Perez A, Vitali GC, Landi F, Genova P (2019). Comparison of different nodal staging in patients with locally advanced mid-low rectal cancer after long-term neoadjuvant chemoradiation therapy. Anticancer Res.

[34] Glynne-Jones R, Counsell N, Quirke P, Mortensen N, Maraveyas A, Meadows HM (2014). Chronicle: results of a randomised phase III trial in locally advanced rectal cancer after neoadjuvant chemoradiation randomising postoperative adjuvant capecitabine plus oxaliplatin (XELOX) versus control. Ann Oncol.

[35] Breugom AJ, van Gijn W, Muller EW, Berglund Å, van den Broek CBM, Fokstuen T (2015). Adjuvant chemotherapy for rectal cancer patients treated with preoperative (chemo)radiotherapy and total mesorectal excision: a Dutch Colorectal Cancer Group (DCCG) randomized phase III trial. Ann Oncol.

[36] Ahn DH, Wu C, Wei L, Williams TM, Wuthrick E, Abdel-Misih S (2017). The efficacy of adjuvant chemotherapy in patients with stage II/III resected rectal cancer treated with neoadjuvant chemoradiation therapy. Am J Clin Oncol.

[37] National Comprehensive Cancer Network (NCCN). NCCN clinical practice guidelines in oncology. https://www.nccn.org/professionals/physician_gls (Accessed on November 09, 2021).

[38] Valentini V, Aristei C, Glimelius B, Minsky BD, Beets-Tan R, Borras JM (2009). Multidisciplinary Rectal Cancer Management: 2nd European Rectal Cancer Consensus Conference (EURECA-CC2). Radiother Oncol.

[39] Zhang C, Xu L, Qin Q, Liu J, Tang X, Jiang N (2019). Good response to neoadjuvant chemoradiotherapy predicts good oncological outcome in locally advanced rectal cancer. Transl Cancer Res.

[40] Xu L, Cai S, Xiao T, Chen Y, Qiu H, Wu B (2017). Prognostic significance of tumour regression grade after neoadjuvant chemoradiotherapy for a cohort of patients with locally advanced rectal cancer: an 8-year retrospective single-institutional study. Colorectal Dis.

[41] Santos MD, Silva C, Rocha A, Matos E, Nogueira C, Lopes C (2013). Tumor regression grades: can they influence rectal cancer therapy decision tree?. Int J Surg Oncol.

[42] Rullier A, Laurent C, Capdepont M, Vendrely V, Bioulac-Sage P, Rullier E (2010). Impact of tumor response on survival after radiochemotherapy in locally advanced rectal carcinoma. Am J Surg Pathol.

